# Avoid, attack or do both? Behavioral and physiological adaptations in natural enemies faced with novel hosts

**DOI:** 10.1186/1471-2148-5-60

**Published:** 2005-11-04

**Authors:** Corinne Vacher, Sam P Brown, Michael E Hochberg

**Affiliations:** 1 Equipe Biologie des Populations en Interaction, Institut National de la Recherche Agronomique (UMR1112), 06903 Sophia-Antipolis Cedex, France; 2 Section of Integrative Biology, University of Texas at Austin, Austin TX 78712, USA; 3 Laboratoire Génétique et Environnement, Institut des Sciences de l'Evolution (UMR5554), Université Montpellier II, 34095 Montpellier Cedex 5, France

## Abstract

**Background:**

Confronted with well-defended, novel hosts, should an enemy invest in avoidance of these hosts (*behavioral adaptation*), neutralization of the defensive innovation (*physiological adaptation*) or both? Although simultaneous investment in both adaptations may first appear to be redundant, several empirical studies have suggested a reinforcement of physiological resistance to host defenses with additional avoidance behaviors. To explain this paradox, we develop a mathematical model describing the joint evolution of behavioral and physiological adaptations on the part of natural enemies to their host defenses. Our specific goals are (i) to derive the conditions that may favor the simultaneous investment in avoidance and physiological resistance and (ii) to study the factors that govern the relative investment in each adaptation mode.

**Results:**

Our results show that (i) a simultaneous investment may be optimal if the fitness costs of the adaptive traits are accelerating and the probability of encountering defended hosts is low. When (i) holds, we find that (ii) the more that defended hosts are rare and/or spatially aggregated, the more behavioral adaptation is favored.

**Conclusion:**

Despite their interference, physiological resistance to host defensive innovations and avoidance of these same defenses are two strategies in which it may be optimal for an enemy to invest in simultaneously. The relative allocation to each strategy greatly depends on host spatial structure. We discuss the implications of our findings for the management of invasive plant species and the management of pest resistance to new crop protectants or varieties.

## Background

In natural antagonistic systems such as host-parasite, plant-herbivore, and predator-prey systems (hereafter called 'host-enemy'), enemies may frequently be confronted with hosts expressing novel defenses. For instance, in agro-ecosystems, herbivorous insects are confronted with novel plant defenses each time a new chemical pesticide or each time a new toxic cultivar is introduced. This situation is frequent in natural areas too, when an herbivorous insect's foraging area is invaded by a novel toxic plant variety or species (*e.g. *invasion of Rocky mountain meadows by *Thlaspi arvense*, a crucifer lethal to *Pieris *larvae [1]). 'Avoid' and 'attack' are two basic strategies that enemies may evolve to cope with these novel defenses. In other words, enemies may evolve the ability to discriminate between defended and undefended hosts and preferentially avoid defended ones (*behavioral adaptation*), or develop a direct counter-adaptation allowing the successful attack of defended hosts (*physiological adaptation*). Both adaptation modes have been extensively studied and reviewed, especially in arthropod systems [2-5].

Although simultaneous investment in both adaptations may first appear to be redundant (since avoiding defended hosts is unnecessary if defenses have been overcome anyway), some empirical studies have suggested a reinforcement of physiological resistance to host defenses with additional avoidance behaviors. The most convincing example is perhaps the study of Pluthero & Threlkeld (1981) [2]. These authors measured the levels of behavioral avoidance and physiological resistance in eight strains of wild-caught *Drosophila melanogaster *tested for their responses to the insecticide malathion. They showed that the most resistant line had also the highest degree of avoidance, suggesting a reinforcement of physiological resistance with an additional avoidance behavior in a wild population. However they did not find any significant correlation between these two modes of insecticide resistance in the eight strains, which indicates that the mechanisms involved were genetically independent from each other. The independence between the two adaptation modes is also suggested by other studies that have demonstrated the evolution of either physiological resistance or behavioral avoidance, but not both. For instance, in *Plutella xylostella*, exposure to transgenic plants expressing toxins from *Bacillus thuringiensis *(*Bt*) induced physiological resistance to *Bt *toxins without discrimination between transgenic and non-transgenic plants [6], whereas exposure to toxic baits altered the behavior of German cockroaches, but not their physiological resistance [7].

To explain the paradox that apparently redundant adaptations may evolve, a number of authors [8-10] have employed population genetics models. These models assume that physiological and behavioral responses are governed by two independent loci each bearing two co-dominant alleles. Nine strategy sets (i.e. nine genotypes) corresponding to the combination of three physiological adaptation levels (high, mild or null) and three behavioral adaptation levels (high, mild or null) are assumed. The results highlight the impact of population-genetic and population-dynamic factors on behavioral and physiological adaptations. In particular, a mixed strategy may be stable depending on the relative fitnesses of the nine genotypes and the initial allelic frequencies [8,9] and the mode of population regulation can have a striking impact on the likelihood of behavioral adaptation to evolve [10]. Simulations by Rausher [10] reveal that under the "hard selection" mode (i.e. regulatory factors act on the population as a whole [11]) the pure behavioral strategy evolves almost as frequently as the pure physiological strategy, whereas under "soft selection" (i.e. the subpopulations of each host are regulated independently [11]) the pure behavioral strategy never evolves. A potential explanation for this difference is that contrary to "hard selection", "soft selection" leads to overcrowding on the most suitable host and undercrowding on the other host [10]. Behavioral generalists therefore have a higher fitness than choosy enemies, because the former suffer lower levels of intra-specific competition. A less frequent outcome (c. 10% of the runs) is the evolution of a mixed strategy (i.e. a strategy where one of the loci at least is polymorphic). Under "hard selection", this outcome is favored by the absence of fitness costs to both traits [10].

The objective of this study is to (i) explore in more detail the conditions favoring the occurrence of a mixed strategy and (ii) study the factors that govern the relative investment into each adaptation mode. Specifically, we study the impact of the shape of the fitness cost functions and the impact of spatial heterogeneity in host defenses. Indeed, while several theoretical models have investigated the impact of the abundance and spatial distribution of suitable hosts on physiological resistance evolution [e.g. [12-17]] or on the evolution of host selection behavior [e.g. [18-21]], the impact of space on the joint evolution of physiological and behavioral adaptations to host defenses has been largely ignored [5]. In our model, physiological resistance and behavioral avoidance are represented as quantitative traits and we adopt an optimality approach to identify the conditions favoring the investments in both traits. Contrary to the population genetics models listed above, this approach permits quantitative predictions of the optimal relative investments in both forms of adaptation. Fitness costs of the adaptations and host spatial structure are assumed to be constant.

Below we show that (i) simultaneous investment may be optimal if the fitness costs of the adaptive traits are accelerating and the probability of encountering defended hosts is low. When (i) holds, we find that (ii) the more that defended hosts are rare and/or spatially aggregated, the more behavioral adaptation is favored.

## Results

### What conditions favor the simultaneous investment in physiological and behavioral adaptations to host defenses?

#### Accelerating fitness costs of adaptations

**Figure 1:**
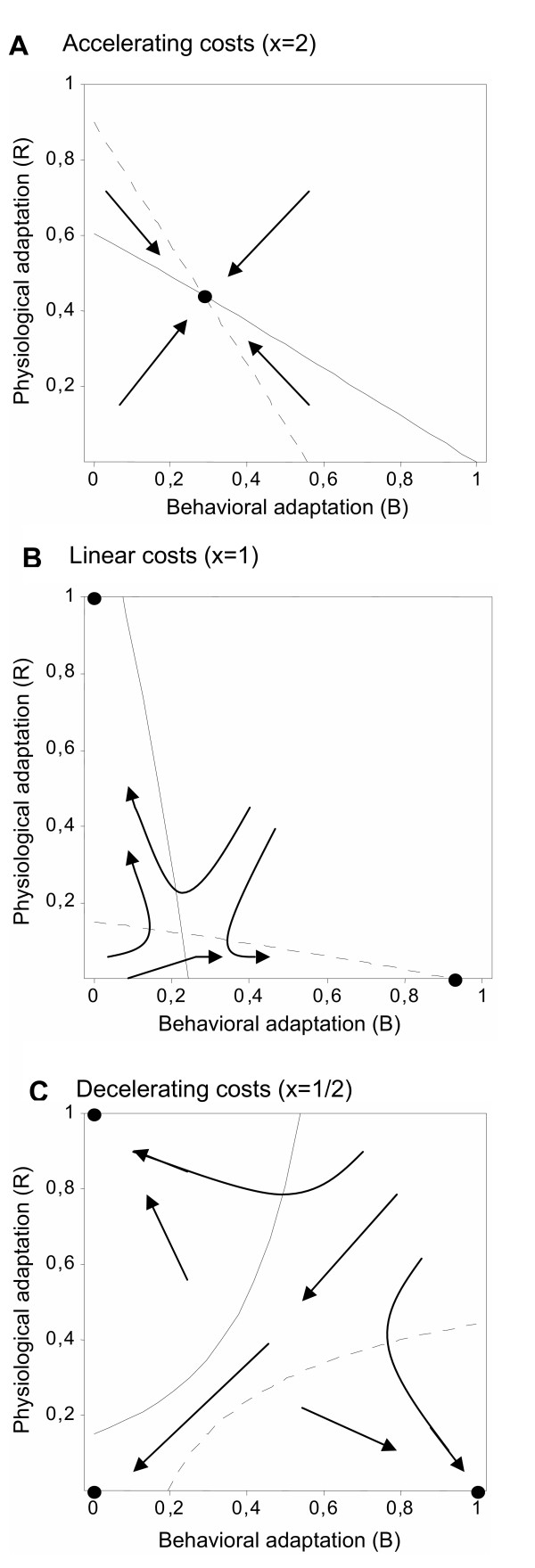
Null clines for physiological (*solid line*) and behavioral (*dotted line*) adaptation to host defenses as a function of the shape of the cost function. Black points are stable steady states. Arrows represent schematic phase trajectories. *k*_*R *_= 0.1, *k*_*B *_= 0.1, *e *= 0.1, *a *= 0.4, *f *= 20%.

We find that the fitness costs of physiological and behavioral adaptations to novel host defenses can have an important effect on the optimal enemy strategy (Fig. 1). In the case of linear and decelerating maintenance costs of adaptations (Fig. 1B and 1C), simultaneous investment in physiological and behavioral adaptation to host defenses is never selected, whatever the spatial configuration of hosts. Either a pure physiological adaptation, a pure behavioral adaptation, or a "no investment" strategy is selected depending on initial investment levels. In the case of accelerating maintenance costs of adaptations (Fig. 1A), we find that a mixed strategy can be optimal.

#### Rare and/or spatially aggregated defended hosts

**Figure 2:**
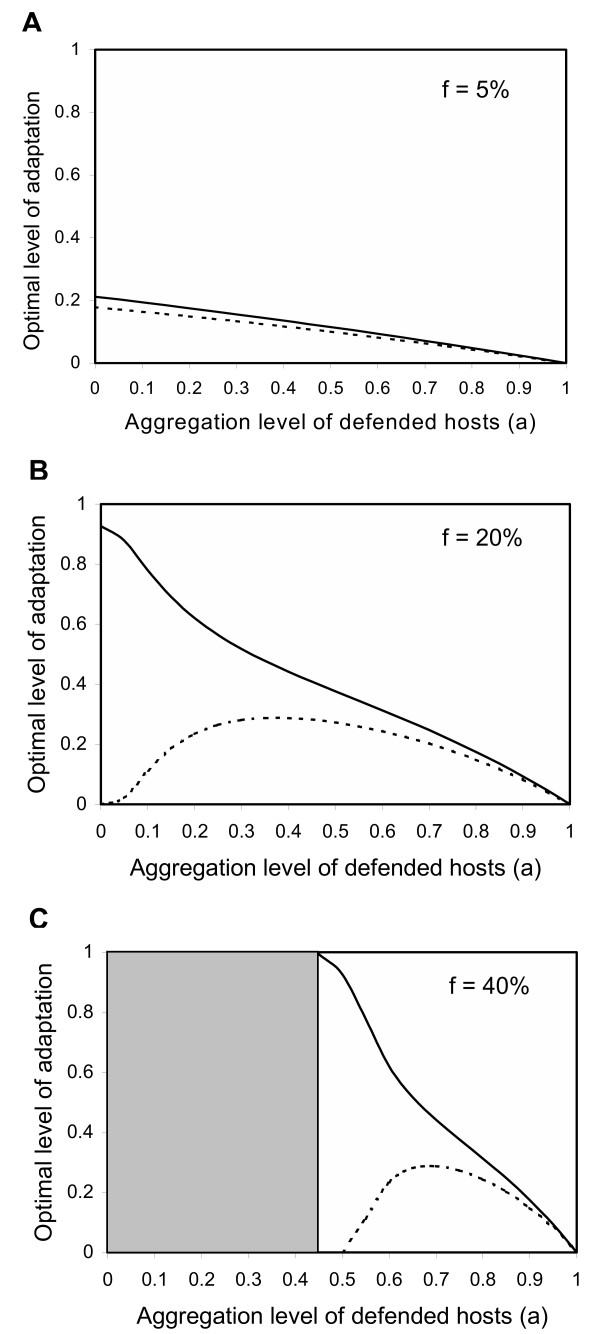
Co-equilibrium (R*, B*) between physiological (*solid line*) and behavioral (*dotted line*) adaptation to host defenses as a function of the frequency *f *and the spatial aggregation level *a *of well-defended hosts. Equilibrium is polymorphic in the white plane and monomorphic in the gray plane ((R*, B*) = (1,0) or (0,1)). *k*_*R *_= 0.1, *k*_*B *_= 0.1, *e *= 0.1, *x *= 2

When considering this latter case in more detail, interestingly, our results show that the spatial configuration of hosts with and without defenses has a strong impact on the occurrence of the mixed strategy (Fig. 2). A mixed investment is only optimal when the frequency of defended hosts in the environment is low or when their aggregation level is high (Fig. 2, white plane). It is also noteworthy that under the canonical set of parameters, the absolute investment in physiological and behavioral adaptations can be very different (Fig. 2B and 2C). In the following, we focus on the factors governing these differences in investment.

### What factors govern the relative investment in each adaptation mode?

#### Spatial configuration of hosts

**Figure 3:**
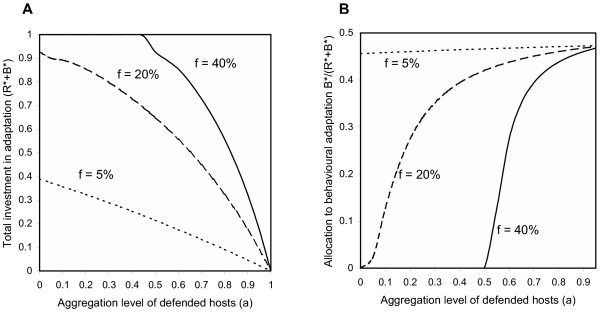
Total investment in adaptation to host defenses (R*+B*) and relative allocation to behavioral adaptation B*/(R*+B*) as a function of the frequency *f *and the spatial aggregation level *a *of defended hosts in the case of a polymorphic equilibrium (R*, B*). Model parameters are the same than in Figure 2.

As Fig. 2 illustrates, investment in each adaptation mode depends on the spatial configuration of hosts. For a given abundance of defended hosts, the absolute level of physiological adaptation is maximal for lower levels of host spatial aggregation than is the absolute level of behavioral adaptation (Fig. 2B: *a *= 0 *vs. a *= 0.4; Fig. 2C: *a *= 0.45 *vs. a *= 0.7). In contrast, looking at the relative investments in each adaptation mode gives a different picture (Fig. 3): a major result of this study is that the relative allocation to behavioral adaptation is maximal when defended hosts are rare and spatially aggregated (Fig. 3B). Under these conditions, we find that the total level of investment approaches zero, because defended hosts are encountered increasingly rarely (Fig. 3A).

#### Magnitude of search costs

**Figure 4:**
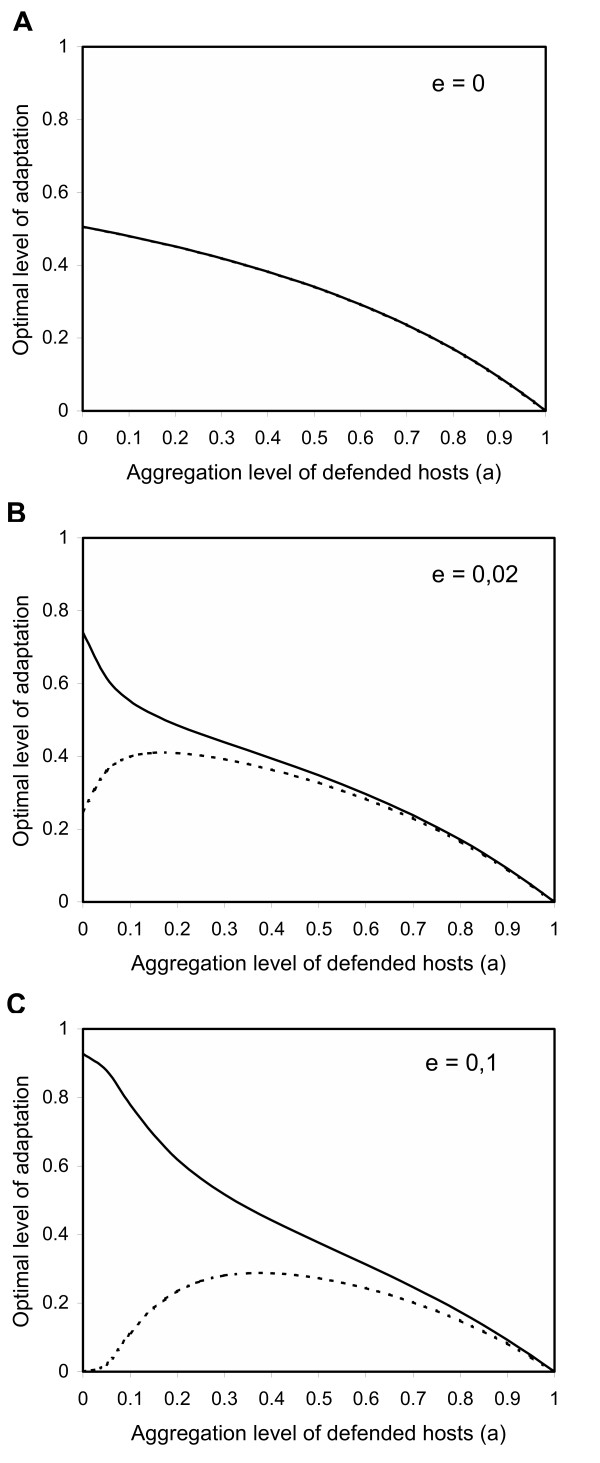
Co-equilibrium (R*, B*) between physiological (*solid line*) and behavioral (*dotted line*) adaptation to host defenses as a function of the search cost coefficient *e *and the spatial aggregation level *a *of well-defended hosts. Equilibrium is polymorphic. *k*_*R *_= 0.1, *k*_*B *_= 0.1, *x *= 2, *f *= 20%

Finally, we investigate the effects of the costs associated with the active search of undefended hosts on the differences in investment in each adaptation mode (Fig. 4). Partial derivatives of enemy fitness with respect to the level of physiological adaptation and the level of behavioral adaptation are symmetric when search costs are zero *(see *Methods, *Eq 7a *and *7b *for *e *= 0). Therefore, under this assumption, optimal investments in each resistance mode are equal (Fig. 4A). Interestingly, the effects of search costs are not uniform across the range of host spatial aggregation levels. Search costs strongly favor physiological over behavioral resistance when defended hosts are randomly distributed, but have a low impact on the relative allocation to each resistance mode when defended hosts are spatially aggregated (Fig. 4B and 4C).

## Discussion

In agreement with previous studies [8-10], we find that the simultaneous investment in avoidance and physiological resistance can be an optimal strategy despite interference between both adaptations (*i.e.*, investment in one adaptation mode decreases the efficiency of investment in the other). We identify two conditions that must be fulfilled: maintenance costs of both adaptations must be accelerating (Fig. 1) and the probability of encountering defended hosts must be low (Fig. 2). Under all other conditions, pure strategies are favored. This parallels the results of Poitrineau and colleagues on host investment in defenses against multiple enemies in the case where defenses interfere with each other [22].

Our results show that in the case where a pure strategy is optimal, *both *the pure physiological resistance strategy and the pure behavioral avoidance strategy may evolve depending on initial investment levels (Fig. 1). This result is in agreement with previous population genetics models that explicitly assume "hard selection" [10], and could be due to our implicit assumption of "hard selection". Indeed, since there is no explicit function of population regulation in our model and because the fitness functions are not dependent on the number or strategy of local competitors, any regulation must occur on the global level.

Moreover, when the mixed investment is optimal, we found that the absolute and relative investments in each adaptation mode are sensitive to the spatial configuration of hosts (Fig. 2). When defended hosts are abundant and/or randomly distributed (i.e., when the probability of encountering defended hosts is high), it is optimal for the enemy to invest mainly in physiological resistance. We have shown that the low investment in behavioral adaptation is due to the costs of actively searching undefended hosts (Fig. 4). As the probability of encountering defended hosts decreases, the absolute investment in behavioral adaptation increases and goes through a maximum. Thereafter, optimal investments in both adaptation modes decrease. In the extreme case, total investment approaches zero because defended hosts are almost never encountered. Looking at the relative investments in each adaptation mode gives a different picture (Fig. 3). Relative investment in physiological resistance is always greater, but the difference tends to disappear with increasing rarity and/or spatial aggregation of defended hosts.

We describe the consequences of interference between two adaptive traits on the joint evolution of these traits in the first part of the Discussion below. In the second and third parts, we highlight the relevance of these findings to plant-herbivore interactions in natural and managed ecosystems.

### Simultaneous investment in avoidance of and resistance to host defenses: a paradox?

Interference between physiological and behavioral adaptations is an emergent property of our model, clearly identifiable from the net fitness benefits of investment in both adaptation modes. Simplifying the enemy fitness *W *for null fitness costs of adaptation (see Methods, Eq. 1a for *k *= *e *= 0) gives

*W *= *W*_0_+*dP*(1+*T*-*I*)

where *T *= *R*+*B *is the total investment in physiological and behavioral adaptations (respectively R and B) and *I *= *BR *is the interference between physiological and behavioral adaptations. Thus, for a given total investment *T*, the net benefits are maximal when all resources are invested in one mode of adaptation only (*i.e.*, interference *I *is zero).

However, the optimality of a strategy also depends on the fitness costs associated with the adaptive traits. Consider an enemy investing an intermediate amount of resources in one adaptation mode and facing an increase in the frequency of encounters with defended hosts. When fitness costs are decelerating, investing in this same mode of adaptation is not only the most efficient but also the least costly way to reinforce adaptation to host defenses. Thus, continuing to invest in the prevailing adaptation mode is better than developing another from zero (*i.e.*, pure resistance strategies are favored). In contrast, for accelerating costs, investing in the prevailing mode of adaptation is more efficient but also more costly. Thus, investing in another adaptation mode can be an optimal choice (*i.e.*, mixed resistance strategies can be optimal). Mixed resistance strategies tend to disappear when the frequency of encounters with defended hosts increases, because investing simultaneously in two interfering adaptive traits is increasingly wasteful. In the extreme case (*R*•1, *B*•1), one half of the investment is useless because of interference.

### Avoidance of aggregated novel hosts: a factor in biological invasions?

Our findings are relevant to adaptation in ecological communities, for example when a habitat is invaded by a plant variety or species that is toxic to a resident herbivore. One commonly observed life history trait in invasive plant species is clonal reproduction [23]. This reproductive mode leads to the spatial aggregation of invaders. Thus, during the initial steps of a biological invasion, clonally invasive plants are rare and spatially aggregated. Our model suggests that natural enemies should invest a low amount of resources into adaptations to these novel hosts, and allocate non-trivial amount of these resources to behavioral adaptation. Consequently, we suggest that selection for avoidance of the toxic compounds produced by rare, clumped invasive plants could be a cause for the so-called "ecological release" experienced by these plants. The enemy release hypothesis states that plant species, on introduction to an exotic region, experience a decrease in regulation by herbivores (in particular, specialist herbivores) and other natural enemies, resulting in a rapid increase in distribution and abundance [24,25]. Comparisons of the parasitic load and the number of pathogens in native versus introduced regions support this hypothesis [26,27], as well as comparison of the plant anti-herbivore compounds [25]. One approach to test our hypothesis would be to compare herbivore behavior in native and introduced ranges of invasive plants.

### Pest management: how to limit physiological resistance to new crop protectants or varieties

Finally, our results are relevant to certain forms of pest management, where one attempts to conserve the efficiency of a new toxic cultivar or a new chemical pesticide. Although models of pest resistance evolution to chemical pesticides or genetically-engineered toxins have long been acknowledged as a tool for pest management [28], host preferences have rarely been incorporated in theoretical developments (*but see *[10,29,30]). Our model suggests that using rare and aggregated treated/toxic plants during the first years of commercialization may curtail a pest's investment in physiological resistance, and favor the evolution of avoidance of treated/toxic areas. By reducing the frequency of encounters with the new pesticide/toxin, this initial step of behavioral adaptation might delay the evolution of physiological resistance if the treated/toxic plants are subsequently used more extensively (*see also *[10]). Refuges (*i.e.*, non-treated/toxic host plants maintained in close proximity to treated/toxic crops to delay physiological resistance evolution [31]) would then serve as insect traps. This potential role of refuges has rarely been studied (*but see *[30]), since in population genetics models refuges are considered to be a source of susceptible insects. Moreover, it is noteworthy that since the commercialization of insect-resistant GM crops, the optimal spatial distribution of refuges for sustainable pest control has received much attention [11-13], but their optimal temporal distribution has rarely been investigated (*but see *[32]). Based on our findings, we suggest that more research should be conducted to define this optimal temporal distribution of refuges, when taking into account the evolution of pest specialization.

## Conclusion

The originality of our study is to have linked together physiology, behavior and landscape structure into a general model describing the adaptation of natural enemies to their hosts. Our model predicts that the optimal strategy for a natural enemy when confronted with well-defended, novel hosts subtly depends on the fitness costs of the adaptations to host defenses and the spatial distribution of defended hosts. Interestingly, under certain conditions (*i.e.*, maintenance costs of the adaptations are accelerating and the probability of encountering defended hosts is low), a reinforcement of physiological resistance to host defenses by avoidance of these same defenses may be optimal. In this latter case, investment in physiological resistance is favored when the novel hosts are abundant at regional scales because the active search of undefended hosts is costly. It is also favored when the host type that might be encountered is difficult to predict by the enemy because the host from which the enemy emerges cannot be used as a cue (*i.e.*, the aggregation level of host types is low).

Although they remain to be confirmed by empirical data, our theoretical results could have important implications for the management of invasive plant species and the management of pest resistance to new crop protectants or varieties. A logical next step in the model analysis would be to enable the host level evolve (*see *[33]). Indeed, since the host level is currently assumed to be non-evolving, our predictions are only relevant to enemy evolution over short time scales (e.g. evolution of herbivorous insects during the initial steps of invasion of their foraging range by new plants) or to systems in which host levels can be managed (e.g. agricultural systems). Relaxing this assumption may be useful to analyze adaptive patterns in coevolving systems having contrasting spatial structures, such as plant-herbivore interactions in tropical and temperate forests [33-36].

## Methods

**Figure 5:**
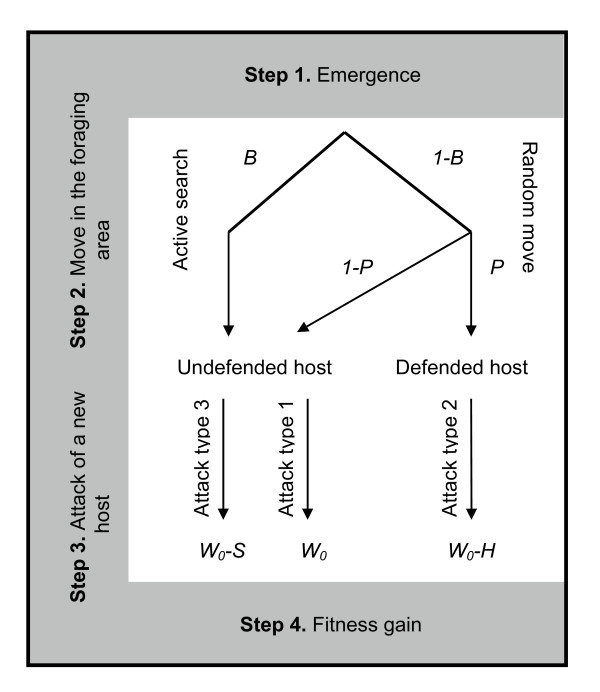
Enemy life cycle. Parameters *W*_0_, *H *and *S *are defined in Table 1.

The model considers a single species of natural enemy confronted with two host types: initial hosts, called 'undefended hosts', and novel hosts, called 'defended hosts'. A simple life-cycle for the enemy that should apply to a range of natural antagonistic systems is assumed: enemies leave their host of emergence (Fig. 5, step 1), engage in a foraging behavior (Fig. 5, step 2) and finally attack a single host individual (Fig. 5, step 3). The host of emergence belongs to the initial host type whereas the selected host may belong to either host type. During the foraging step (Fig. 5, step 2), enemies either move at random over their environment or engage in the active search of undefended hosts. Enemies can therefore adopt three types of offensive behavior (Fig. 5, step 3):

1. attack of an undefended host randomly encountered in the foraging area

2. attack of a defended host randomly encountered in the foraging area

3. attack of an undefended host encountered by active search in the foraging area.

Physiological and behavioral adaptations to host defenses are assumed to be quantitative traits that respectively decrease the deleterious effects of host defenses during a type (2) attack and increase the frequency of type (3) attacks.

The enemy's fitness *W *is stated as

*W *= *W*_G_*(1-C_*R*_)*(1-C_*B*_*)     (1a)*

where *W*_*G *_is a function of *R *and *B *representing the average fitness gain resulting from host attack and *C*_*R *_and *C*_*B *_are increasing functions of *R *and *B *representing the fitness costs of adaptations. The average fitness gain resulting from host attack *W*_*G *_is stated as

*W*_*G *_= *(1-B)(1-P)W*_0_*+ (1-B)P(W*_0_*-H) +B(W*_0_*-S)     (1b)*

where the three terms respectively reflect fitness gains resulting from attacks of type (1), (2) and (3). Each term is detailed below.

The first term corresponds to the attack of an undefended host encountered by a random move in the foraging area. The fitness gain resulting from this type of attack equals *W*_0_, which is the maximal potential fitness gain resulting from a host attack. The probability of this event is *(1-B)(1-P)*, where *B *is the probability for an enemy to engage in the active search of undefended hosts during the foraging step and *P *is the probability for an enemy to randomly encounter a defended host in its foraging area (Fig. 5). *B *corresponds to the investment in behavioral adaptation whereas *P *depends on the spatial configuration of defended and undefended hosts. This latter is described by the frequency *f *of defended hosts and their level *a *of spatial aggregation at the scale of an enemy's foraging range, which is the scale of spatial aggregation the most relevant to our study [37-39]. Host aggregation at the scale of the enemy's foraging range is described by the average frequencies of the three possible host-pair types (*i.e. *defended-defended, undefended-defended and undefended-undefended), when considering only host-pairs between which distance is inferior to the maximal foraging distance [40]. The higher the frequency of homologous pairs, the greater the spatial aggregation. Making a parallel with Wright's inbreeding coefficient in population genetics [41], we define the aggregation level *a *of defended hosts at the scale of the enemy's foraging range as the deficit in heterologous host pairs. Defended-defended, undefended-defended and undefended-undefended host-pair average frequencies, *f*_*dd*_, *f*_*ud *_and *f*_*uu *_respectively, are defined as

*f*_*dd *_= *f*^*2*^+*af(1-f) *    *(2a)*

*f*_*ud *_= *2f(1-f)(1-a) *    *(2b)*

*f*_*uu *_= *(1-f)*^*2*^+*af(1-f). *    *(2c)*

The mean probability *P *that an enemy emerging from an undefended host encounters a defended host after a random move in its foraging area is

*P *= *1/2 f*_*ud*_*/(f*_*uu *_+ *1/2 f*_*ud*_*)     (3a)*

which simplifies to

*P *= *f(1-a). *    *(3b)*

The second term of equation (1b) corresponds to the attack of a defended host encountered by a random move in the foraging area. The probability of this event is *(1-B)P. *The fitness gain *W*_0 _is decreased by *H, H *being the fitness loss due to the deleterious effects of host defenses (Fig. 5). *H *is assumed to decrease with the level *R *of physiological enemy resistance and increase with the level *d *of host defense. We chose the simplest function to describe *H *[42]. Hence

*H *= *d(1-R). *    *(4)*

The third term of equation (1b) corresponds to the attack of an undefended host encountered by active search. The probability of this event is *B. *The fitness gain *W*_0 _is decreased by *S, S *being the fitness loss due to active searching [43,44] (Fig. 5). *S *is assumed to increase with the probability *P *of encountering a defended host by any random move. We also chose the simplest function to describe *S*. Hence

*S *= *eP *    *(5)*

where *e *is the search cost coefficient.

Let us now describe the constitutive fitness costs of adaptations, *C*_*R *_and *C*_*B *_(Eq. 1a). Evidence of fitness costs of adaptation to host defenses is scarce [45] and a fortiori, the shape of the fitness cost functions (i.e., variations in the cost magnitude with the level of investment in the adaptive trait) is largely unknown [22,45]. However, it is reasonable to assume that physiological and behavioral adaptations to host defenses have constitutive fitness costs: a few studies show that physiological resistance to toxic compounds results from permanent metabolic changes that can reduce fitness [47-49] and obviously, discrimination between defended and undefended hosts involves energy allocation to sensors and neural cells allowing the detection and treatment of signals. Consequently, we chose simple functions to describe the constitutive fitness costs of physiological and behavioral adaptations to host defenses and we assessed the robustness of model predictions to the shape of these functions. The cost functions are taken as

*C*_*R *_= *k R*^*x *^    *(6a)*

*C*_*B *_= *k B*^*x *^    *(6b)*

where *k *is the cost coefficient and *x *controls the form of the function. If *x *> 1 then the cost is accelerating, if *x *= 1 then it is linear, whereas if *x *< 1 then it is decelerating.

Finally, note that we assume that the two adaptive traits are independent [8-10,50]. Partial derivatives of enemy fitness *W *(Eq. 1a) with respect to the level of physiological adaptation *R *and the level of behavioral adaptation *B *give

*∂W/∂R *= *(1-C*_*B*_*)((1-B)dP(xC*_*R*_+*R(1-C*_*R*_*-xC*_*R*_*))-xC*_*R*_*W*_0_*)/R + ePB(1-C*_*B*_*)xC*_*R*_*/R *    *(7a)*

*∂W/∂B *= *(1-C*_*R*_*)((1-R)dP(xC*_*B*_*+B(1-C*_*B*_*-xC*_*B*_*))-xC*_*B*_*W*_0_*)/B - ePB(1-C*_*R*_*)(1-(1+x)C*_*B*_*)/B *    *(7b)*

The optimal strategy (R*, B*) is assessed based on the position of the null clines ∂*W/∂R = 0 *and ∂*W/∂B = 0 *[51]. All the analyses were done with Mathematica 4 [52]. All the model parameters are summarized in Table 1.

**Table 1:** Model parameters, their definitions, range of values employed, and notes on their use.

**Parameter**	**Definition**	**Range**	**Comments**
**Host**			
d	Defense level of defended hosts	Held at 1	
f	Frequency of defended hosts in the environment	0–1	
a	Spatial aggregation level of defended hosts	0–1	a = 0 when defended and undefended hosts are randomly distributed and a→1 when defended and undefended hosts form two distinct patches.
**Enemy**			
P	Probability of encountering a defended host during a random move in the foraging area	0–1	Increases with the frequency f of defended hosts and decreases with their aggregation level a P = f(1-a)
W_0_	Maximal potential fitness gain when attacking an host	Held at 1	Corresponds to the fitness gain when attacking a randomly encountered undefended host
H	Fitness loss due to host defense	0–1	Decreases with the level of physiological resistance R of the enemy and increases with the level of defense d of the host H = d(1-R)
S	Fitness loss due to active searching of undefended hosts	0–1	Increases with the difficulty in finding undefended hosts, i.e., with the probability P of encountering a defended host during a random move S = eP
e	Search cost coefficient	0–1	
R	Physiological adaptation level	0–1	Physiological adaptation reduces the fitness loss H when attacking a defended victim.
C_R_	Physiological adaptation maintenance cost	0–1	Increases with the level of physiological adaptation R C_R _= kR^x^
B	Behavioral adaptation level	0–1	Behavioral adaptation corresponds to the probability of engaging in the active search of undefended hosts.
C_B_	Behavioral adaptation maintenance cost	0–1	Increases with the level of behavioral adaptation B C_B _= kB^x^
k	Maintenance cost coefficient	0–1	
x	Shape coefficient of the maintenance cost functions	Held to 1/2, 1 or 2	Maintenance costs increase with the level of adaptation in an accelerating (x = 2), linear (x = 1) or decelerating (x = 1/2) way

## Authors' contributions

CV and SPB conceived the study and designed the model. CV performed the model analysis and wrote the paper. MEH coordinated the study and obtained funding to finance the research. All authors read and commented on drafts of the manuscript, and approved the final manuscript.
